# Multimodal management of a large-volume brainstem hemorrhage with stereotactic drainage, targeted antimicrobial therapy, and early neurorehabilitation: a case report

**DOI:** 10.3389/fnins.2025.1719867

**Published:** 2025-12-18

**Authors:** Xinyuan Han, Zhijun Huang, Jing Ning, Wenhui Shang

**Affiliations:** Department of Neurological Rehabilitation, Shaanxi Provincial Rehabilitation Hospital, Xi’an, China

**Keywords:** brainstem hemorrhage, case report, hematoma puncture drainage, multidrug-resistant organisms infection, neurorehabilitation

## Abstract

Moderate-volume brainstem hemorrhage (>5 mL) typically carries an extremely poor prognosis. This article presents a rare case of survival following a large-volume brainstem hemorrhage with therapeutic intervention. A 47-year-old male with hypertension presented with sudden-onset coma. CT imaging revealed a 10.3 mL brainstem hemorrhage. The patient underwent hematoma puncture drainage and tracheostomy, accompanied by hemostatic therapy, intracranial pressure reduction, anti-infection treatment, and neurorehabilitation. During treatment, the patient developed recurrent multidrug-resistant bacterial infections. After 2 months, the patient gradually regained consciousness with improved limb muscle strength, though exhibiting motor aphasia, dysphagia, and urinary/fecal incontinence. Rehabilitation therapy was continued. This case demonstrates that even with large-volume brainstem hemorrhage, comprehensive management including timely drainage, intracranial pressure control, tracheostomy, infection management, and early rehabilitation may achieve survival and consciousness recovery. However, as a single-case report, this study has limited sample size, necessitating further large-scale randomized controlled trials to validate these findings.

## Introduction

Primary brainstem hemorrhage accounts for approximately 5–10% of spontaneous intracerebral hemorrhages, with prognosis strongly correlated to hematoma volume. Even a minimal volume of brainstem hemorrhage can lead to severe consequences (Zheng et al., 2022). Studies indicate that patients with hemorrhage volumes exceeding 5 mL face mortality rates as high as 60–80%, and survivors often suffer severe neurological deficits (AlMohammedi et al., 2020; Wang et al., 2019; Zheng et al., 2022). The optimal management of moderate-volume (5–10 mL) brainstem hemorrhage remains controversial, though emerging evidence suggests potential benefits from early surgical intervention (Chen et al., 2021; Ding et al., 2024; Pustilnik et al., 2024). We present a rare case of a hypertensive patient with a 10.3 mL brainstem hemorrhage who achieved survival and consciousness recovery following hematoma puncture drainage and comprehensive medical therapy. This case highlights the critical role of managing complications, particularly multidrug-resistant bacterial infections (Wang et al., 2023), and provides valuable insights into the treatment of large-volume brainstem hemorrhages.

## Case description

**FIGURE 1:**
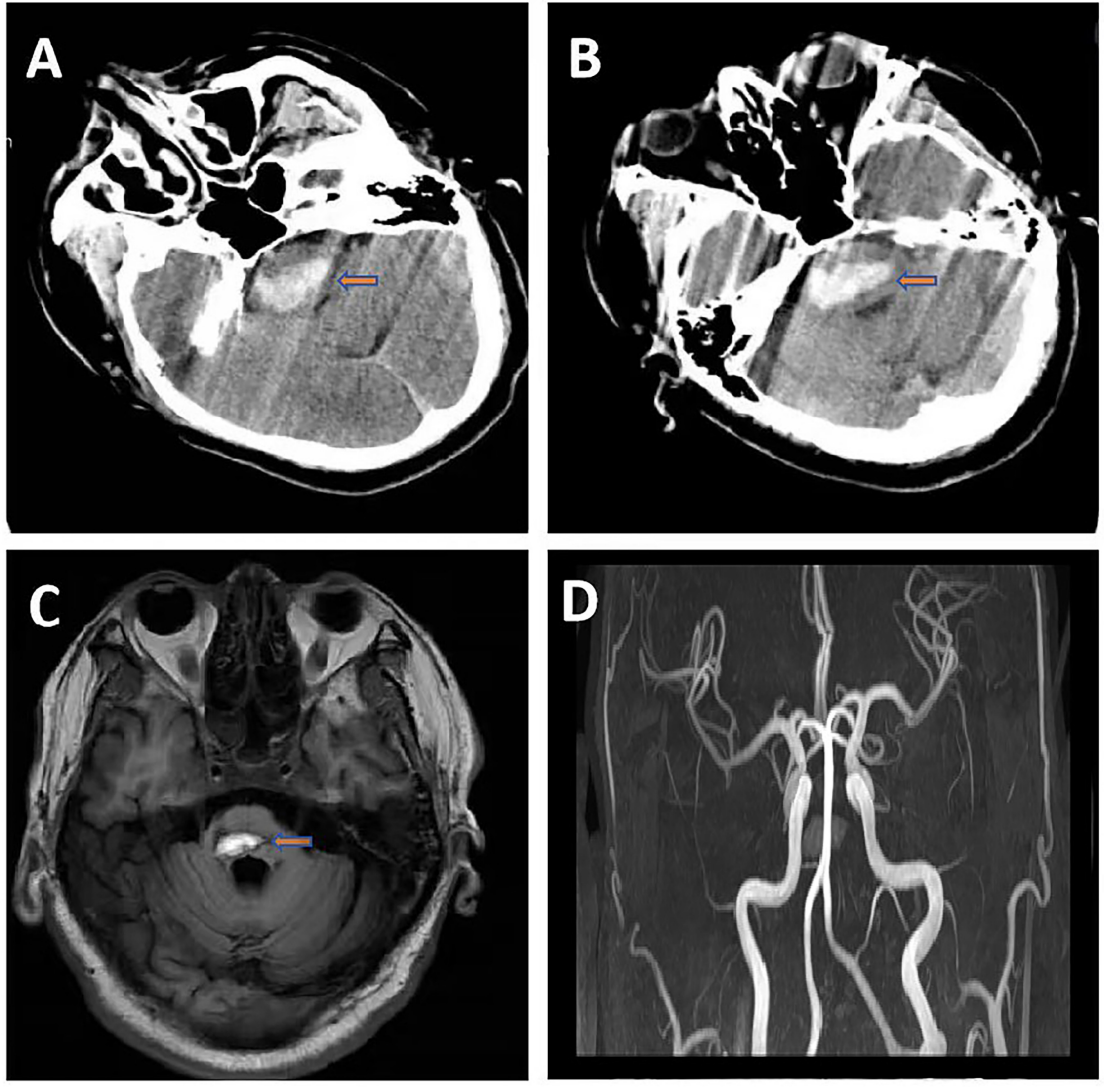
**(A,B)** Cranial computed tomography showed high-density shadow in the brainstem (volume 10.3 ml, CT value 53.6 Hu) with surrounding edema (volume 5.2 ml, CT value 33.9 Hu) consistent with acute brainstem hemorrhage. **(C)** Cranial magnetic resonance imaging showed hypointense ring-like signal around the lesion on T2-weighted images, indicating chronic phase changes of brainstem hemorrhage in the right paramedian region. **(D)** Cranial magnetic resonance angiography showed the bilateral internal carotid arteries, middle cerebral arteries, anterior cerebral arteries, basilar artery, and major branches of the posterior cerebral arteries have a natural course, with no stenosis or deformation.

A 47-year-old male with a decade-long history of poorly controlled hypertension (non-compliant with medication) and significant tobacco exposure (26 pack-years), along with a positive family history of hemorrhagic stroke (maternal death from intracerebral hemorrhage). He was transported to our emergency department and admitted within 1 h of symptom onset, presenting with sudden coma and agonal respiration. Neurological examination revealed deep coma (Glasgow Coma Scale score of 6) with bilateral isocoric pupils (2 mm in diameter) demonstrating intact light reflexes, along with generalized hypotonia. Emergency cranial computed tomography (CT) demonstrated a primary brainstem hemorrhage measuring 10.3 mL (53.6 Hounsfield units) with surrounding edematous changes (5.2 mL, 33.9 Hounsfield units) (Figures 1A, B). The patient underwent emergent stereotactic-guided hematoma evacuation followed by tracheostomy. Postoperative management included 72-hour tranexamic acid administration, a stepwise mannitol protocol for intracranial pressure control, and comprehensive metabolic support. Within 24 h postoperatively, the patient developed a significant increase in sputum production (>150 mL/day). Sputum culture identified multidrug-resistant Acinetobacter baumannii (MDR-AB). Initial antimicrobial susceptibility testing demonstrated sensitivity to both tigecycline (minimum inhibitory concentration [MIC] = 2 mg/L) and cefoperazone-sulbactam (MIC = 16/8 mg/L). A targeted combination regimen was immediately initiated, consisting of intravenous tigecycline (100 mg loading dose, followed by 50 mg every 12 h) plus cefoperazone-sulbactam (3 g every 8 h, calculated based on the sulbactam component). This regimen was maintained for 2 weeks, during which the infection was initially controlled. In the third postoperative week, the patient experienced recurrent fever (peak 39.7 °C), and chest CT revealed bilateral lower lobe consolidations. Repeat sputum culture confirmed persistent colonization by the same MDR-AB strain, but with a significant shift in the susceptibility profile: the meropenem MIC increased from 4 mg/L (intermediate) to 16 mg/L (resistant), while the isolate remained susceptible to polymyxin B (MIC = 1 mg/L). Accordingly, the antimicrobial therapy was escalated to polymyxin B (loading dose 2.5 mg/kg, then 1.5 mg/kg every 12 h) in combination with meropenem (2 g every 8 h, administered as a 3-hour extended infusion). This adjusted regimen was administered for a total of 2 weeks, leading to infection control. This combination was selected to exploit the potential synergistic effect between polymyxin B and meropenem against the resistant Acinetobacter baumannii isolate. Throughout the clinical course, a comprehensive bedside neurorehabilitation protocol was implemented, including diaphragmatic pacing, swallowing and speech therapy, passive limb mobilization, functional electrical stimulation, and traditional acupuncture.

By the two-month follow-up, the patient had regained consciousness but exhibited significant neurological sequelae, including motor aphasia, severe dysphagia (requiring nasogastric tube feeding), and neurogenic bladder/bowel dysfunction (managed with indwelling catheterization). Neurological examination demonstrated essentially normal muscle tone. Motor examination revealed proximal upper extremity strength of Medical Research Council Manual Muscle Testing scale (MRC) grade III, hand grip strength grade I, and lower extremity strength grade II, though he retained the ability to communicate purposefully through head nodding/shaking. Respiratory status remained compromised, with persistent tracheostomy dependence and tenacious secretions requiring frequent suctioning (>10 times daily). Follow-up neuroimaging demonstrated near-complete resolution of the brainstem hemorrhage on Magnetic Resonance Imaging (MRI) (Figure 1C), with Magnetic Resonance Angiography (MRA) showing no evidence of vascular malformations or aneurysms (Figure 1D). While hemodynamically stable, the patient continued to demonstrate severe functional impairment (modified Rankin Scale score 5), necessitating ongoing intensive rehabilitation and comprehensive airway management.

The timeline of systematic nursing interventions and monitoring throughout the described clinical course is presented (detailed in Table 1).

**TABLE 1:** Timeline of key nursing interventions and clinical correlations.

Time phase	Core nursing interventions	Objective and clinical correlation
Acute phase (within 24 h post-op)	Continuous vital signs and neurological monitoring (q1–2 h) Initiation of airway management and sputum characteristic monitoring	Early detection of postoperative complications Establishing foundational airway care
Early infection phase (post-op day 1)	Strict documentation of sharp increase in sputum production (>150 mL/day) Immediate sputum culture collection and submission Enhanced airway humidification and postural drainage	Initial identification of objective evidence for pulmonary infection Providing microbiological basis for early MDR-AB diagnosis Improving pulmonary secretion clearance
Persistent infection phase (post-op days 2–20)	Continued monitoring of sputum volume and characteristics Administration of tigecycline + cefoperazone-sulbactam Maintained intensive airway care	Evaluating initial antibiotic regimen effectiveness Ensuring accurate implementation of first-line targeted therapy
Exacerbated infection phase (post-op week 3)	Close monitoring of temperature fluctuations (peak 39.7 °C) Enhanced respiratory management (suctioning >10 times/day) Coordination of chest CT examination	Identifying objective evidence of infection progression Managing deteriorating respiratory function Providing imaging basis for antibiotic regimen adjustment
Recovery phase (≥2 months post-onset)	Continued nasogastric tube management and oral care Standardized management of neurogenic bladder Implementation of graded rehabilitation training	Ensuring long-term nutritional support Preventing long-term complications Promoting neurological function recovery

This table systematically demonstrates how nursing monitoring facilitated early infection detection, guided antibiotic selection, evaluated treatment efficacy, and supported clinical decision-making, highlighting the critical role of nursing in infection identification and management.

## Discussion

Brainstem hemorrhage is a life-threatening condition with a high mortality rate (Gao et al., 2024), particularly when the hematoma volume exceeds 5 mL (Teo et al., 2023; Yu et al., 2023). In this case, a 47-year-old male patient with hypertension experienced a brainstem hemorrhage of 10.3 ml. Survival and recovery of consciousness were achieved through a combination of stereotactic hematoma puncture drainage and comprehensive management, challenging the prognosis of large-volume brainstem hemorrhages. The favorable outcome of this case relied on the following key strategies.

Timing and value of minimally invasive intervention. Early (<6 h) hematoma puncture drainage significantly reduced secondary brainstem injury. Studies confirm that stereotactic puncture can lower intracranial pressure and improve cerebral perfusion in patients with brainstem hemorrhage, significantly increasing survival rates compared to conservative treatment (Pustilnik et al., 2024). In this case, the patient’s Glasgow Coma Scale score improved from 6 to full consciousness postoperatively, highlighting the importance of timely decompression for neuroprotection. Consistent with the conclusion that microsurgical procedures reduce mortality rates (Chen et al., 2021; Du et al., 2022; Zhou et al., 2023).

Precise control of Multidrug-Resistant Organisms infections. Postoperative Multidrug-resistant Acinetobacter baumannii (MDR-AB) pneumonia was managed using a stepwise anti-infective regimen (tigecycline polymyxin B + meropenem) (Tamma et al., 2024). Research shows that MDR-AB infections are a major risk factor for death in neurocritical patients (Černiauskienė and Vitkauskienė, 2025; Diao et al., 2024), and combined therapy based on antibiotic susceptibility testing can increase the cure rate of pulmonary infections (Shi et al., 2019).

Necessity of early multimodal rehabilitation. Neurorehabilitation interventions, including motor therapy, bedside diaphragmatic pacing, functional electrical stimulation, and acupuncture, were initiated during the acute phase. This approach aligns with the concept of early rehabilitation (Langhorne and Ramachandra, 2020; Liu et al., 2014; Marek et al., 2024). The early rehabilitation protocol promoted neuroplasticity through synergistic multimodal sensory input and targeted functional stimulation. Repetitive limb exercises and functional electrical stimulation, adhering to the Hebbian plasticity principle, strengthened synaptic connections between the sensorimotor cortex and the corticospinal tract. Concurrently administered swallowing training and diaphragmatic pacing provided rhythmic patterned input, activating brainstem swallowing centers and respiratory rhythm generators respectively. Combined with traditional acupuncture’s modulation of the neural microenvironment, this integrated intervention created essential conditions for neural network reorganization across synaptic, circuit, and system levels, providing a mechanistic basis for consciousness recovery and partial functional improvement. Studies indicate that early pulmonary rehabilitation following intracerebral hemorrhage can reduce the incidence of pneumonia and improve respiratory function (Troosters et al., 2023; Winstein et al., 2016; Yang et al., 2024). In the present case, the persistent need for tracheostomy and suctioning even after infection control underscores the requirement for prolonged airway management in cases of severe brainstem injury (Hansen et al., 2023).

Although the patient’s modified Rankin Scale score remained 5 at 2 months, Cranial MRI showed almost complete absorption of the hematoma and no vascular malformations, laying the foundation for future rehabilitation.

In summary, this case suggests that minimally invasive surgery to alleviate primary injury, personalized anti-infection control of complications, and early rehabilitation to promote neural remodeling are the three essential elements in treating large-volume brainstem hemorrhage. This integrated management protocol contrasts with previous studies that focused predominantly on isolated interventions, employing either hematoma puncture drainage alone or conventional medical management (Ding et al., 2024; Sun et al., 2025), whereas our therapeutic approach achieved survival and consciousness recovery in a patient with hemorrhage exceeding 10 mL through systematic integration of three essential elements. It is important to acknowledge the inherent limitations of a single-case report. The favorable outcome observed in this patient could be influenced by confounding factors, including his biological reserve (e.g., younger age) and the precise location of the hematoma within the brainstem, which might have spared critical nuclei and tracts. Therefore, a direct causal attribution of survival and consciousness recovery solely to our multimodal intervention cannot be definitively established.

## Data Availability

The original contributions presented in this study are included in this article/supplementary material, further inquiries can be directed to the corresponding author.
